# Association between 2D:4D ratios and sprinting, change of direction ability, aerobic fitness, and cumulative workloads in elite youth soccer players

**DOI:** 10.1186/s13102-023-00654-y

**Published:** 2023-03-28

**Authors:** Hadi Nobari, Özgür Eken, Pablo Prieto-González, Rafael Oliveira, João Paulo Brito

**Affiliations:** 1 Department of Exercise Physiology, Faculty of Educational Sciences and Psychology, University of Mohaghegh Ardabili, Ardabil, 56199-11367 Iran; 2 Faculty of Sport Sciences, University of Extremadura, Cáceres, 10003 Spain; 3 Department of Physical Education and Sport Teaching, Inonu University, Malatya, Turkey; 4 Health and Physical Education Department, Prince Sultan University, Riyadh, 11586 Saudi Arabia; 5 Sports Science School of Rio Maior–Polytechnic Institute of Santarém, Rio Maior, 2040-413 Portugal; 6 Research Center in Sport Sciences, Health Sciences and Human Development, Vila Real, 5001-801 Portugal; 7 Life Quality Research Centre, Rio Maior, 2040-413 Portugal

**Keywords:** Football, Young players, Maturation, Training load, Performance

## Abstract

**Background:**

The aim of this study was two-fold: (i) to determine the correlation between 2D:4D, maximal oxygen uptake (VO_2max_), body fat percentage (BF%), maximum heart rate (HRmax), change of direction (COD), and accumulated acute and chronic workload variables; (ii) to verify if the length of the second digit divided by fourth digit (2D:4D) can explain fitness variables and accumulated training load.

**Methods:**

Twenty elite young football players (age: 13.26 ± 0.19 years; height: 165.8 ± 11.67 cm; body mass: 50.70 ± 7.56 kg; VO_2max_, 48.22 ± 2.29 ml.kg^− 1^.min^− 1^) participated in the present study. Anthropometric and body composition variables (e.g., height, body mass, sitting height, age, BF%, body mass index, right and left finger 2D:4D ratios) were measured. The following fitness tests were also conducted: 30 − 15 Intermittent Fitness Test (VO_2max_ and HRmax), COD (5-0-5 agility test), and speed (10-30msprint test. HRmax and the training load were also measured and monitored using the Rate of Perceived Exertion during the 26 weeks.

**Results:**

There were associations between HRmax and VO_2max_, between 2D and 4D lengths and Left and Right hand ratios. Also, in AW with Right and Left 4D. The CW and de ACWR with the Right 4D. There were other associations between physical test variables and workload variables.

**Conclusions:**

Under-14 soccer players with low right and left-hand 2D:4D ratios did not perform better in the selected fitness tests to assess VO_2max_, COD, or sprint ability. However, it cannot be ruled out that the absence of statistically significant results may be related to the small sample size and the maturational heterogeneity of the participants.

## Introduction

Young soccer players tended to cover about ~ 3965–6500 meters of total distance and ~ 12–250 m of high-speed running (> 18 km.h^-1^) in training sessions (data until under-19 players) [1] while n matches, the total distance can achieve ~ 2038–11734 meters, and high-speed running (> 18 km.h^-1^) ~ 230–976 meters (data until under-20 players) [2]. These distances included soccer-specific skills and abilities such as change of direction (COD) that when performed at high intensities, can lead to injuries, particularly in young soccer players, due to the changes associated with growth and maturity levels [3]. For this reason, it is essential to monitor those levels. In this regard, a significant and inexpensive marker of athletic performance is the digit ratio (2D:4D) which is calculated using the length of the second digit (2D) divided by the length of the fourth digit [4].

In some sports (e.g., soccer, basketball, rowing, athletics), negative associations were found between the 2D:4D and strength test results [19–21]. In under-17 (U-17) young soccer players, a recent study analyzed the associations of 2D:4D ratio on variations of accumulated workloads and fitness parameters. The study confirmed that 2D:4D was a relevant predictor of physical fitness (i.e., VO_2max_) and changes in young soccer players [5]. Nevertheless, to our knowledge, the previous variables have not been used in U-14 soccer players. However, for young soccer players (particularly those U-14), the information available about using ratios is still scarce.

Moreover, when devices such as global positioning systems are not available, one way to control the training load could be the rating of perceived exertion (RPE), which can also be multiplied by session duration to generate the session-RPE (s-RPE) [6, 7]. These measures allow for calculating other ratios, such as the acute: chronic workload ratio (ACWR) [8–10]. The mentioned ratio uses the accumulated load during one week (acute load) and the load of the past four weeks (chronic load) to understand their relationship [8]. It also allows obtaining more knowledge about the players´ status for a better training design.

In addition to these assessments, evaluating aerobic fitness is necessary to acknowledge the physical condition of the athletes [11, 12] and to adjust the training intensity to the player´s current level of performance. For this purpose, some variables are used to quantify the performance, such as those related to running/sprinting and acceleration activities and COD movements. These actions are associated with explosive movements and anaerobic capacity [13]. Thus, assessing such characteristics is essential to improve the performance of young soccer players. One of the most commonly used tests to determine this condition is the 30 − 15 Intermittent Fitness Test (30-15IFT) since it presents good sensitivity [14] and is suitable for soccer training due to its intermittent nature. This test was designed to obtain the final velocity at 30-15IFT to prescribe high-intensity interval training better. Additionally, it is helpful to predict VO_2max_ [15]. Another essential ability in soccer is COD. It is generally associated with a change of speed which can contribute to a decisive moment in the match and ultimately score a goal [16].

Therefore, the objective of this study was two-fold: (i) Estimate the correlation between 2D:4D, VO_2max_, body fat percentage (BF%), maximum heart rate (HR_max_), COD, and accumulated workload (AW) and chronic workload (CW) variables; (ii) Verify if 2D:4D can explain fitness variables and AW. It is hypothesized that players with low right and left hand 2D:4D would perform better [17]. It was also hypothesized that 2D:4D ratio would predict the changes in the previous variables in the soccer players [17, 18].

## Material and method

### Experimental approach to the problem

This investigation was conducted as a prospective study with a cohort observational methodology. It was performed on a cross-sectional basis where researchers followed the players throughout the season, and evaluations were conducted once. This experiment was conducted over a duration of 26 weeks. RPE was collected daily during this period, and AW and accumulated chronic workload (CW) were calculated. One set of anthropometric measurements—including height and the ratio of 2D:4D— was taken in the first week. These measurements were made in the morning [19, 20]. After these measurements, the following evaluations were performed on each particular day: COD, 10 m and 30 m sprinting maximum, and afterwards, VO_2max_.

## Subjects

The sample for this study consisted of 20 elite young football players ( chronological age: 13.26 ± 0.19 years; height: 165.8 ± 11.67 cm; body mass: 50.70 ± 7.56 kg; VO_2max_, 48.22 ± 2.29 ml.kg^− 1^.min^− 1^). The inclusion criteria were: (a) U-14 age category players; (b) having competed first in the regional league and, subsequently, in the national league; (c) participated in at least 90% of their training seasons; (d) participated in weekly soccer matches while the payers that participated in separate training sessions was considered the exclusion criteria.

We calculated the sample size based on the following variables with G-Power software model 3.1.9.2 (University of Düsseldorf, Düsseldorf, Germany) software: a priori, correlation: point biserial model method, high correlation size between 2D:4D ratios, bio motor abilities parameters, and workload parameters in youth soccer players according to previous studies [21–23]; α err prob = 0.05, and power (1-β err prob) = 0.90. The analysis determined that 20 participants have a statistical power of more than 90.38%.

Prior to the start of this research, written permission was obtained from parents and athletes. The present research was approved by the Ethical Committee of the University of Mohaghegh Ardabili ethics and was also conducted in agreement with the principles established in the declaration of Helsinki.

## Data measurement and variables

### Anthropometric and finger measurements

Measurements were performed in the morning period between 08:30 a.m. to 10:30 a.m., since all participants were instructed to be in a fasted state with an empty bladder and without doing exercise in the previous 24h. To measure 2D and 4D finger lengths [4], the participants placed their right and left hands on the scanner (5590 HP Scanjet, Hewlett-Packard, Palo Alto, California, USA), keeping a distance between their index and middle fingers of two centimetres. After transferring the palm image from the scanner to the computer, the researcher measured the finger lengths using the Kinovea software (version 0.8.15). From the basal (flexion) proximal phalanx to the distal phalanx, the 2D and 4D finger lengths were determined. The ratio of the two fingers was determined by dividing 2D by 4D. The right finger 2D:4D ratio (RF 2D:4D) minus left finger 2D:4D ratio (LF 2D:4D) was used to determine the difference, which was defined as Dif-L-F ratio [24].

Finally, the same observer evaluated intra-observer reliability twice. The evaluation was done one week apart. The 2D:4D ratio had an intraclass correlation coefficient (ICC) between 0.96 and 0.98.To determine the standing height, participants stood barefoot in the stadiometer. Then, participants´ heels, hips, shoulder blades, back, and head were held as close as possible to the stadiometer, being the feet positioned side-by-side. Next, the participants sat on a 50 cm bench, brought their buttocks as close as possible to the stadiometer with a straight back, and placed their hands on their feet. In this position, their heights were measured. Sitting height was estimated as the distance between the highest point of the head and the bench. For this measurement, a portable SECA (Model 213, Germany) stadiometer with a 5 mm precision was used [25]. The UK-made Seca model 813, with an accuracy of 0.1 per kg, was utilized to measure the body mass. The measurements were taken following the norms of the international organization for the improvement of kinanthropometry (ISAK) [26], and they were performed in the morning prior to breakfast [19].

## Sprint test

For the sprint test, a digital timer coupled to two photocells was mounted at hip height, and following a 10-minute specialized warm-up, participants stood 70 centimeters in front of the starting line. To determine the sprint time, two tests were conducted at a distance of 10 and 30 meters [27]. The evaluation system was carried out using FitLight Trainer® sensors (Fitlight Sport Corp., Ontario, Canada). The timing gates were lowered to a suitable hip height after considering the average height of the participants in the sample group. The time recorded for each participant was saved on a portable tablet equipped with an Android operating system, and its subsequent analysis was performed in the application Microsoft Windows® Excel (Redmond, Washington, USA). The best result out of three trials was utilized for statistical analysis. Subjects were required to rest for at least 3 minutes between attempts. The coach monitored all aspects of the examination. In this assessment, the ICC obtained was 0.87.

## COD test

The 5-0-5 test was used for the COD ability [28]. As a result, this entailed performing a 10-meter linear sprint after a stationary start, completing a 180-degree turn on a predefined turn leg (right or left) while maintaining contact with a designated line, and then making a 5-meter return sprint while crossing an established finish line. The time it took to run the final five meters of the 10-meter linear sprint, turn around, and run the final five meters back was timed and recorded [29]. The speed assessment phase includes two attempts with a 2-minute rest break between each repetition. Then, the results of each trial were averaged, and they were used in the subsequent analysis. The time was measured in seconds. The COD test was applied for both right foot (COD-R) and left foot (COD-L).

## Intermittent fitness test 30–15

30-15IFT was utilized to calculate VO_2max_. It was conducted on a soccer field. This test consists of repeated 2x20-m runs forward and back between the starting, turning, and finishing line (equal 40-m shuttle) at a progressively increased speed controlled (0.5 km.h^-1^ in each stage) with a beep sound, with the average speed beginning at 8 km.h^-1^ [15]. After the first beep, the athlete starts running at 8 km.h^-1^ for 30 seconds; between each running bout. Then, they rest for 15 seconds, and the test continues until they reach volitional exhaustion or fail to come on three consecutive occasions to the 3-m lines. The score registered was the speed at the participants’ last stage (VIFT). Then, VO_2max_ was calculated according to the following formula [30]: VO_2max_ (ml.kg^–1^.min^–1^) = 28.3 – [2.15 x G (sex)] – [0.741 x A (age)] – [0.0357 x W (weight)] + (0.0586 x A x VIFT) + [1.03 x VIFT (final running speed)], being VIFT the subjects’ final speed in the test of exhaustion. Before the test, all competitors performed warm-ups under a strength and conditioning coach’s guidance. The ICC for this test was 0.92 [5]. HRmax was measured with a Mi-Band 3 that each participant wore on the wrist during 30-15IFT. The HRmax recorded was that obtained at the end of the test.

### Monitoring internal training loads

A CR-10 Borg’s scale (category-ratio-10 Borg´s scale) was used to record the RPE [31, 32]. Before conducting the present research, the players were already familiar with the scale since they had been using the RPE for at least three years. To determine the athlete’s total training load, the validity and reliability of this scale have been confirmed by previous research [7, 33, 34]. Responses to the question “How intense was your session?” were structured on a scale ranging from 0 to 10 arbitrary units (A.U.), being 0 minimum effort and 10 maximal efforts. Players responded to the abovementioned questions half an hour after completing the training session. In addition, the total amount of time spent in the training sessions was recorded (in minutes). The session RPE (s-RPE) was calculated to determine the level of internal load by multiplying the score on the CR-10 scale by the total number of minutes spent in the session [31, 35]. In this study, the accumulated load refers to the total workload over 26 weeks, including training and competition.

#### Calculation of Acute: chronic workload

Additional calculations were made with RPE. The total daily training load throughout the week was deemed the weekly AW. It was monitored the team’s average weekly AW throughout all 26 weeks of the season and documented (acWL). The formula shown below was used to estimate the CW. The following equation was used to determine each player’s CW [34, 36]:

*CW* = (*AWn* − 1 + *AWn* − 2 + *AWn* − 3) × 0.333 where n = week number.

The variable ACWR was used to determine and record the mean ratio of the team’s accumulated workload (AW) members to their CW members. The following formula was utilized to determine the ratio of the individual AW to the individual CW [36], where n = week number.

$$\varvec{A}\varvec{C}\varvec{W}\varvec{R}=\frac{\varvec{A}\varvec{W}\varvec{n}}{(\varvec{A}\varvec{W}\varvec{n}-1 + \varvec{A}\varvec{W}\varvec{n}-2 + \varvec{A}\varvec{W}\varvec{n}-3) \times 0.333 }$$

### Statistical analysis

Statistical analyses were performed using GraphPad Prism 8.0.1 (GraphPad Software Inc, San Diego, California, USA). Data are presented as mean and standard deviation (SD). Shapiro–Wilk was applied to check the normality of the data. After its confirmation, Pearson correlation analysis was performed between 2D:4D, VO_2max_, BF%, HRmax, COD, sprint, AW and CW. The following ranges were considered for the correlation coefficient sizes: <0.1 = trivial; 0.1–0.3 = small; > 0.3–0.5 = moderate; > 0.5–0.7 = large; > 0.7–0.9 = very large; and > 0.9 = nearly perfect correlation [37]. To model the relationship between variables, a linear regression analysis was used. VO_2max_, VIFT, COD right and left foot, HRmax, BF%, 10 m, and 30 m were set as predictor variables and LF-2D:4D and RF-2D:4D ratios as dependent variables. The significance level was set at p < 0.05.

## Results

Table 1a presents the results of the finger measurements, Table 1b the results of the fitness tests and training load parameters, Table 2 shows the correlation between 2D:4D and sprint, COD, aerobic fitness, Table 3 shows the correlation between 2D:4D and cumulative WL parameters.

**Table 1:** **a.** Finger measurements

Variables	Mean ± SD	Confidence Interval 95%
RF-2D (cm)	7.2 ± 0.4	6.4 to 8.0
RF-4D (cm)	7.2 ± 0.4	6.3 to 8.3
Dif-R-L-Ratio	1.0 ± 0.0	0.9 to 1.0
LF-2D (cm)	7.2 ± 0.4	6.3 to 8.0
LF-4D (cm)	7.2 ± 0.4	6.3 to 8.3
Dif-L-F-Ratio	1.0 ± 0.0	0.9 to 1.1
LF-2D:4D	1.0 ± 0.0	0.9 to 1.0
RF-2D:4D	1.0 ± 0.0	0.9 to 1.0

RF-2D: Length of the digit two-finger of the right hand; RF-4D: Length of the digit four-finger of the right hand; Dif-R-L-Ratio: Difference right-fingers; LF-2D: length of the digit two-finger of the left hand; LF-4D: Length of the digit four-finger of the left hand; Dif-L-R-Ratio: Difference left-fingers; LF-2D:4D: Ratio minus left-fingers 2D:4D ratio; RF-2D:4D: Ratio minus right-fingers 2D:4D ratio.

**Table 1:** **b.** Study participants results obtained in the selected assessments

Variables	Mean ± SD	Confidence Interval 95%
10 m sprint (sec)	1.2 ± 0.09	1.1 to 1.4
30 m sprint (sec)	3.6 ± 0.20	3.4 to 4.01
COD right foot	2.1 ± 0.1	1.9 to 2.6
COD left foot	2.1 ± 0.1	1.9 to 2.4
HRmax (bpm)	202.7 ± 10.7	170.0 to 214.0
RPE (A.U.)	4.0 ± 0.1	3.1 to 4.8
s-RPE (A.U.)	301.2 ± 73.02	52.23 to 395.8
All-Training duration (min)	7354 ± 1453	1310 to 8210
Average- Training duration (min)	75.81 ± 14.98	13.51 to 84.63
ACWR (A.U.)	0.9 ± 0.0	0.8 to 1.0
acWL (A.U.)	32,719 ± 2675	25,605 to 37,010
AW (A.U.)	1285 ± 68.1	1157 to 1423
CW (A.U.)	1283 ± 73.5	1149 to 1458
VIFT	16.43 ± 1.47	13.50 to 18.50

HRmax: Maximum heart rate max; COD: Change of direction; RPE: Rate of perceived exertion; s-RPE: session rating of perceived exertion; ACWR: Acute chronic workload ratio; acWL: Accumulated workload training; AW: Average of accumulated workload training; CW: Accumulated chronic workload in the season; A.U: Arbitrary units.

**Table 2:** Correlation between 2D:4D and sprint, COD, aerobic fitness

Variables	β0	β1	β2	β3	β4	β5	β6	β7	β8	β9	β10	β11	β12	β13	β14	β15	β16	β17	β18
VO_2max (_β0)	1																		
HRmax (β1)	0.498*	1																	
BF% (β2)	-0.183	-0.299	1																
Right 2D (β3)	-0.12	0.178	0.071	1															
Right 4D (β4)	-0.037	0.197	0.086	**0.742**#	1														
Dif-L-R ratio (β5)	-0.1	-0.002	-0.045	0.376	-0.34	1													
Left 2D (β6)	-0.117	0.224	0.035	**0.992**£	**0.719**§	0.4	1												
Left 4D (β7)	0.02	0.196	0.088	**0.736**§	**0.984**£	-0.326	**0.714**§	1											
Dif-L-F ratio (β8)	-0.193	0.04	-0.064	0.352	-0.331	**0.956**£	0.393	-0.36	1										
COD R foot (β9)	-0.068	0.1	0.135	0.041	0.253	-0.288	-0.002	0.179	-0.236	1									
COD L foot (β10)	-0.341	-0.346	-0.023	0.224	0.29	-0.091	0.186	0.199	-0.014	0.229	1								
LF-2D:4D (β11)	-0.107	-0.01	-0.025	0.365	-0.351	**0.998**£	0.389	-0.336	**0.953**£	-0.304	-0.114	1							
RF-2D:4D (β12)	-0.17	0.055	-0.074	0.361	-0.325	**0.961**£	0.403	-0.351	**0.998**£	-0.244	-0.035	**0.959**£	1						
10 m (β13)	-0.063	-0.048	0.437	0.134	0.242	-0.142	0.074	0.16	-0.111	**0.721**§	0.42	-0.15	-0.11	1					
30 m (β14)	-0.059	-0.188	0.348	-0.293	0.019	-0.423	-0.339	-0.075	-0.34	**0.556**#	0.238	-0.427	-0.34	**0.771§**	1				
RPE (β15)	0.253	-0.189	0.362	-0.088	0.074	-0.215	-0.091	0.14	-0.285	-0.083	-0.079	-0.203	-0.288	0.097	0.04	1			
s-RPE (β16)	0.306	-0.248	0.342	-0.071	0.017	-0.119	-0.093	0.051	-0.175	-0.05	-0.07	-0.106	-0.177	0.052	0.053	**0.599#**	1		
TD-top (β17)	0.282	-0.196	0.223	-0.042	-0.03	-0.018	-0.067	-0.03	-0.043	-0.01	-0.027	-0.008	-0.04	0.062	0.062	0.239	**0.915£**	1	
TD-average (β18)	0.282	-0.196	0.223	-0.042	-0.03	-0.018	-0.067	-0.03	-0.043	-0.01	-0.027	-0.008	-0.04	0.089	0.089	0.04	**0.915£**	**1.000**£	1

Significant differences (p ≤ 0.05) are highlighted in bold; HRmax: Heart rate max; BF%: body fat percentage; 2D: Second digit; 4D: Fourth digit; Dif-R-L-Ratio: Difference right-fingers; Dif-L-R-Ratio: Difference left-fingers; COD R foot: Change of direction right foot; COD L foot: Change of direction left foot; LF-2D:4D: Ratio minus left-fingers; RF-2D:4D: Ratio minus right-fingers; TD-top: Total time duration; TD-average: Average time duration; s-RPE: Session rating of perceived exertion; *: Moderate effect; #: Large effect.; §: Very large effect; £: Nearly perfect effect

**Table 3:** Correlation between 2D:4D and cumulative WL parameters

Variables	β1	β2	β3	β4	β5	β6	β7	β8	β9	β10	β11	β12	β13	β14
Right 2D (β1)	1.000													
Right 4D (β2)	**0.742**#	1.000												
Dif-L-R ratio (β3)	0.376	-0.340	1.000											
Left 2D (β4)	**0.992**£	**0.719**§	0.400	1.000										
Left 4D (β5)	**0.736**§	**0.984**£	-0.326	**0.714**§	1.000									
Dif-L-F ratio (β6)	0.352	-0.331	**0.956**£	0.393	-0.360	1.000								
LF-2D:4D (β7)	0.365	-0.351	**0.998**£	0.389	-0.336	**0.953**£	1.000							
RF-2D:4D (β8)	0.361	-0.325	**0.961**£	0.403	-0.351	**0.998**£	**0.959**£	1.000						
AW (β9)	0.316	**0.500**#	-0.244	0.312	0.456*	-0.161	-0.259	-0.185	1.000					
ACWR (β10)	-0.281	− 0.467*	0.250	-0.277	-0.422	0.166	0.264	0.189	**-0.992**£	1.000				
CW (β11)	0.258	0.450*	-0.257	0.258	0.404	-0.167	-0.271	-0.191	**0.987**£	**-0.997**£	1.000			
TD-top (β12)	-0.042	-0.030	-0.018	-0.067	-0.030	-0.043	-0.008	-0.040	0.331	-0.313	0.289	1.000		
TD-average (β13)	-0.042	-0.030	-0.018	-0.067	-0.030	-0.043	-0.008	-0.040	0.331	-0.313	0.289	**1.000**£	1.000	
acWL (β14)	0.150	0.123	0.031	0.168	0.101	0.100	0.024	0.079	**0.844**§	**-0.846**§	**0.842§**	**0.534**#	**0.534**#	1.000

Significant differences (p ≤ 0.05) are highlighted in bold; 2D: Second digit; 4D: Fourth digit; Dif-R-L-Ratio: Difference right-fingers; Dif-L-R-Ratio: Difference left-fingers; LF-2D:4D: Ratio minus left-fingers; RF-2D:4D: Ratio minus right-fingers; AW: Accumulated acute workload in the season; CW: Accumulated chronic workload in the season; RPE: Rate of perceived exertion; ACWR: Acute chronic workload ratio; TD-top: Total time duration; TD-average: Average time duration; acWL: Accumulated workload training; *: Moderate effect; #: Large effect.; §: Very large effect; £: Nearly perfect effect

As for the association between variables, as shown in Table 2 and Table 3, neither Dif-L-R ratio nor Dif-L-F ratio, or LF-2D:4D or RF-2D:4D maintained statistically significant correlations with the following variables: VO_2max_, HRmax, BF%, COD right and left foot, 10 m, 30 m, TD-top and TD-average.

**Fig. 1:**
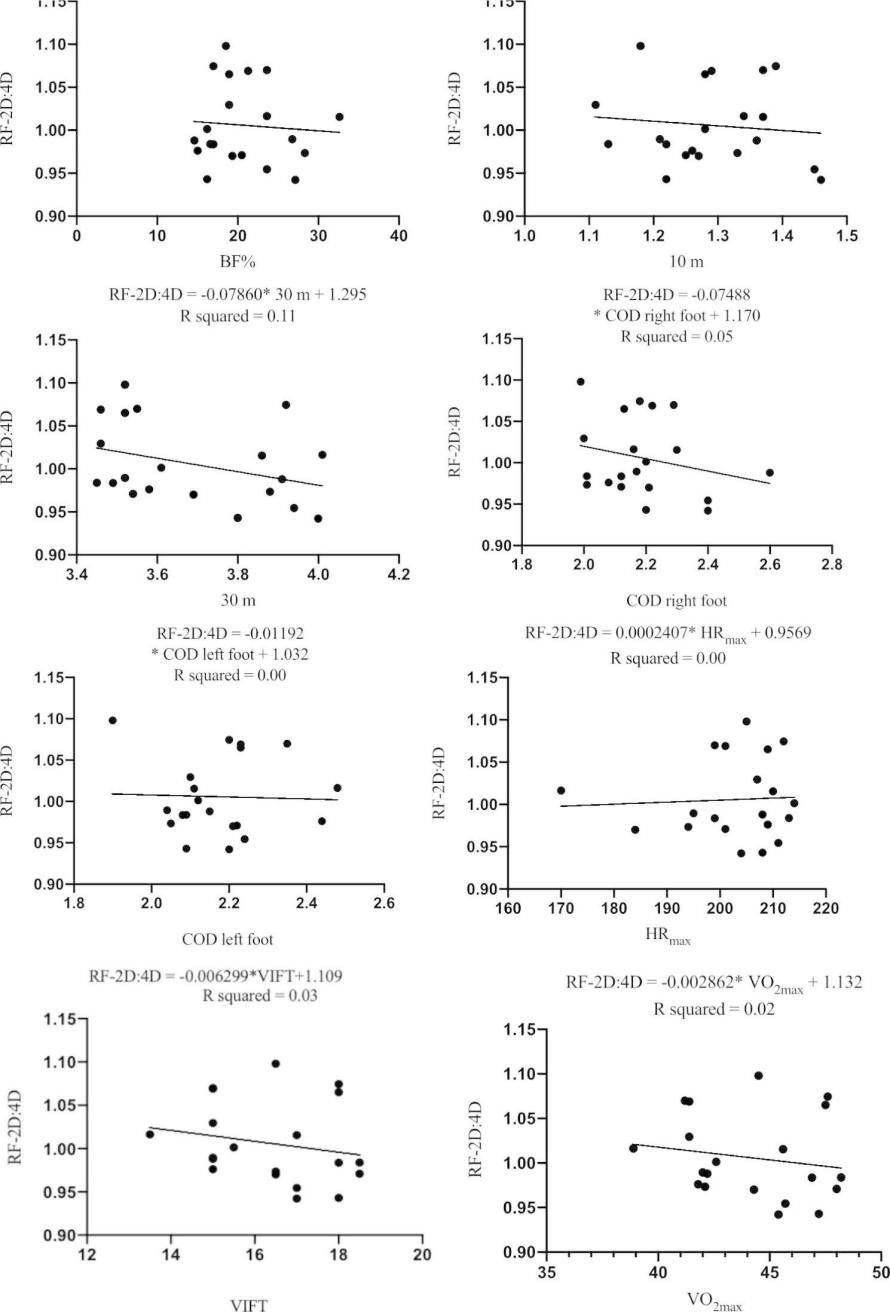
Linear regression analysis: Percentage of variation between RF-2D:4D and VO_2max_, VIFT, COD right and left foot, HRmax, BF%, 10 and 30 m variables

Figure 1 shows the results of linear regression analysis conducted to explain the relationship between potential predictor variables (VO_2max_, VIFT, COD right and left foot, 10 m, and 30 m) with RF-2D:4D ratio. However, the coefficients found were not statistically significant (all, p > 0.05).

**Fig. 2:**
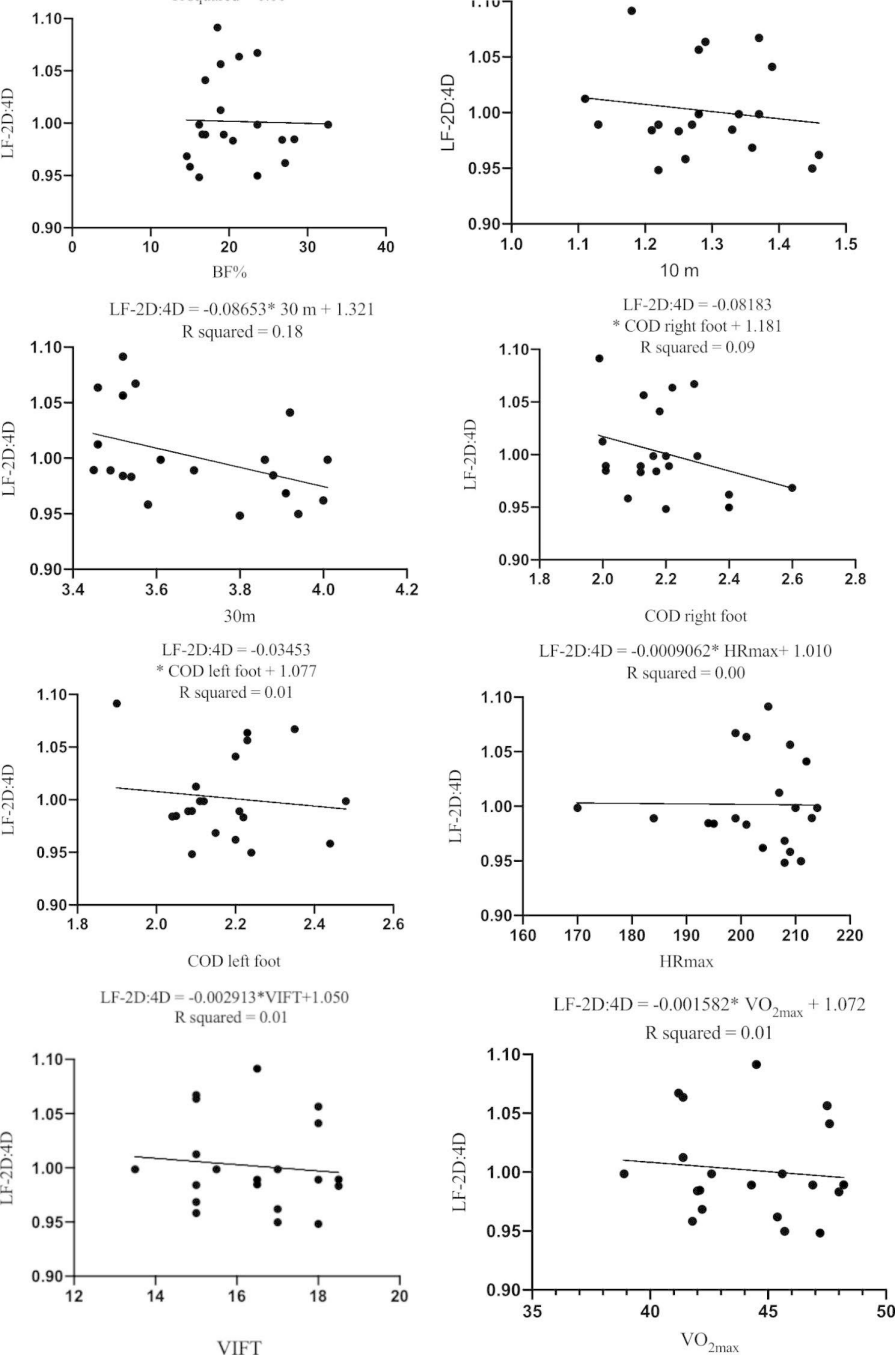
Linear regression analysis: Percentage of variation between LF-2D:4D and VO_2max_, VIFT, COD right and left foot, HR_max_, BF%, 10 m, and 30 m variables

In Fig. 2, the results of linear regression analysis conducted to explain the relationship between potential predictor variables (VO_2max_, VIFT, COD right and left foot, 10 m, and 30 m) with LF-2D:4D ratio are present. However, the coefficients obtained were not statistically significant (all, p > 0.05).

## Discussion

The aims of this study were: (i) determine the correlation between 2D:4D, VO_2max_, BF%, HRmax, COD, AW, and CW variables; and verify if 2D:4D ratio could explain fitness variables and AW. According to the results, there were no significant associations between physical performance markers and the 2D:4D ratio. In addition, this ratio could not explain fitness variables and AW.

The predictive validity of physical parameters on game performance has been demonstrated at university and professional levels [5, 13, 38, 39]. In this sense, physical assessment in youth soccer should be considered a valuable tool to improve talent identification. However, the present study had no associations between 2D:4D ratio and athletic performance markers. We consider that these results may be due to the study participants´ age (U14), since there is a great maturational heterogeneity at this stage. This may prevent finding statistically significant findings [38], mainly when the sample size is small.

Several studies [40–42] have highlighted a set of variables that can influence athlete development and sport skill acquisition. There are also variables such as quantity and quality of training [43] or psychological factors such as motivation or resilience [44] to consider, and other less studied factors such as the cut-off dates used for creating age groups [45]. The present study found no relationship between the biomarker 2D:4D ratio and the total daily training load of the week, the cumulative load of four weeks, and the ACWR (determined for 26 weeks). However, over the past decade, researchers have hypothesized that finger length, especially the ratio between the second and fourth digits, is a biomarker of prenatal testosterone exposure, a factor proposed to predict latent talent for sports [4, 46]. As the ratio of the individual AW to the individual CW was determined, it would be expected that players with the smaller ratio (considered an indicator of greater testosterone exposure) would perform better in ACWR. This hypothesis is based on the fact that greater testosterone levels improve performance due to the positive effect on endurance-related parameters [43].

The digit ratios found in the present study (right hand, 1.0 ± 0.0; left hand, 1.0 ± 0.0) were higher than those of Baker et al. [43] with 240 male handball players of similar age (right hand, 0.963 ± 0.032; left hand, 0.983 ± 0.031). Values of less than 1 reflect a more extended ring finger than an index finger (or a more ‘‘masculinized’’ digit ratio) [47]. Thus, we can speculate that our sample is still in the peri-pubertal phase, although the sample size is too small compared with the previous study.

The 2D:4D ratio effects may be ‘’strong’’ enough to explain general differences in performance/attainment between extreme groups (e.g., athletes and non-athletes) [43], but insufficient to explain subtle differences between those selected at a single stage of development. Moreover, predictors of performance are primarily unstable across product [48]. Some authors reported significant differences between different maturity offset groups (-2.5, -1.5, -0.5, 0.5, 1.5, and 2.5 years from peak height velocity) found in neuromuscular power tests such as the vertical jump and 10m and 20m run [49].

As for the body mass analysis, the standard deviation was about 15% of the sample body mass. This may largely determine upper body strength and power and justify the lack of association between variables. According to previous research [38], more mature players could outperform all other players except those at 1.5 years from the age of PHV. However, the authors reported that prepubertal showed better VO_2max_ than other groups (i.e., the pubertal and post-pubertal). In contrast, another study conducted with basketball athletes aged between 14–15 showed that the post-pubertal subjects performed better than the pubertal and pre-pubertal individuals in Yo-Yo IR1 (770.7 > 613.1 > 430.9 m) [50].

Considering the present and previous research findings, there is a disparity of results regarding the relationship between the results obtained in different physical fitness tests and athletic performance markers, such as 2D:4D ratio. Although it is thought to be related to diverse gender, phenotypes, and hormone-related traits, including sports ability, results have often been conflicting and based on small sample sizes [51]. For this reason, further research with better test standardization procedures and more significant sample sizes is warranted.

This study possesses both notable strengths and limitations. In terms of resilience, the findings offer coaches valuable insights into the assessment and monitoring of soccer players, particularly considering the absence of associations and predictive capabilities of 2D:4D with other variables. However, it is important to acknowledge certain limitations. Firstly, the participants were exclusively drawn from one team, potentially limiting the generalizability of the results. Additionally, the sample size employed in this study was relatively small, and future investigations should incorporate larger sample sizes to enhance the reliability of the findings. Furthermore, as suggested by prior research [5], longitudinal studies may yield more robust and comprehensive outcomes and should be conducted in the future to build upon these findings.

## Conclusions

Contrary to existing evidence indicating that 2D:4D ratios are good performance markers in individual and team sports, the present study verified that U14 soccer players with low right and left-hand 2D:4D ratios do not show higher performance in selected fitness tests. Similarly, the 2D:4D ratio did not help predict motor performance in tests commonly used by soccer players to estimate their VO_2max_, COD, or sprinting ability. However, it cannot be ruled out that the absence of statistically significant results may be related to the small sample size and the study participants’ maturational heterogeneity.

## Data Availability

The data presented in this study are available on request from the corresponding author.

## Abbreviations

VO_2max_: Maximal oxygen uptake
BF%: Body fat percentage
HRmax: Maximum heart rate
COD: Change of direction
30-15IFT: 30 − 15 Intermittent Fitness Test
RPE: Rating of perceived exertion
s-RPE: Session rating of perceived exertion
ACWR: Acute:chronic workload ratio
2D: Length of the second digit
4D: Length of the fourth digit
2D: 4D:length of the second digit divided by fourth digit
AW: Accumulated workload
CW: Chronic workload
RF: Right finger
LF: Left finger
ICC: Intraclass correlation coefficient
A.U.: Arbitrary units
VIFT: Speed at the participants’ last stage
SD: Standard deviation
acWL: Total of 26 weeks of accumulated workload training.
